# Condition-dependent effects of knockdown of autophagy on *C. elegans* longevity

**DOI:** 10.1101/2025.07.28.667102

**Published:** 2025-07-31

**Authors:** Kuei Ching Hsiung, Hannah Chapman, Xiaoya Wei, Xiaoyu Sun, Isadora Rawlinson, David Gems

**Affiliations:** Institute of Healthy Ageing, and Research Department of Genetics, Evolution and Environment, University College London, London, UK.

**Keywords:** aging, condition dependence, germline, insulin/IGF-1 signaling, nematode

## Abstract

Autophagy is thought to clear damaged cellular constituents that contribute to aging, and several life-extending interventions in model organisms show some degree of autophagy dependence. In *C. elegans*, inhibiting autophagy can shorten, lengthen or have no effect on lifespan. Differences between published findings likely reflect variability in experimental conditions. Here we investigate the condition dependence of effects on lifespan of RNA-mediated interference (RNAi) knockdown of autophagy pathway components. Effects on several interventions causing a strong Age (increased lifespan) phenotype were examined, including mutation of *daf-2* (insulin/IGF-1 receptor). Factors varied included *daf-2* mutant allele class, *atg* gene, temperature and presence of 5-fluoro-2’-deoxyuridine (FUDR). Effects on lifespan of *atg* RNAi proved to be highly condition dependent. Notably, for most *atg* genes tested lifespan was not usually reduced more in the long-lived mutant than in the wild-type control. This occurred at 20°C for certain *atg* genes with *daf-2(e1368)* but not *daf-2(e1370)*. At 25°C, little reduction in lifespan was seen. However, *atg-18* knockdown behaved differently, suppressing *daf-2* Age under all conditions, suggesting possible pleiotropic action. Presence of high concentration FUDR caused knockdown of several *atg* genes to increase lifespan. Thus, depending on experimental conditions, *atg* knockdown can increase, decrease or have no effect on *daf-2* Age. The lack of suppression of Age by *atg* RNAi in most cases raises questions about the importance of autophagy in *daf-2* Age. Moreover, condition dependence of effects creates a risk of possible condition selection bias.

## Introduction

A long-standing theory is that senescence (aging) is largely a consequence of the accumulation of random molecular damage caused by, among other things, reactive oxygen species (Beckman and Ames, 1998; Harman, 1956; Murphy, 2023; Shore and Ruvkun, 2013; Szilard, 1959). This view predicts that mechanisms of somatic maintenance, particularly those that prevent accumulation of damaged cellular constituents, will decelerate the aging process. One somatic maintenance function viewed as a potential longevity-assurance process is autophagy (specifically macroautophagy), which effects lysosome-dependent degradation of cellular constituents, including damaged matter (Aman et al., 2021).

The possible anti-aging role of autophagy has been extensively tested in the short-lived nematode *Caenorhabditis elegans*, and supporting evidence found with respect to several life-extending interventions, including reduced insulin/IGF-1 signaling (IIS), reduced germline signaling, and dietary restriction (Hansen et al., 2018). These studies were principally performed in the 2000s; however, during the same period, falsification tests of the molecular damage theory, particularly the oxidative damage theory, led to some uncertainty about its validity (Gems and Doonan, 2009; Perez et al., 2009; Shields et al., 2021). Meanwhile, an alternative theoretical framework emerged, based on the evolutionary theory of aging (Arnold and Rose, 2023; Williams, 1957), arguing that senescence is largely the consequence of genetically-determined, programmatic mechanisms (Blagosklonny, 2006; de Magalhães and Church, 2005; Gems, 2022; Maklakov and Chapman, 2019), and very much so in *C. elegan*s (Gems and de la Guardia, 2013; Gems et al., 2021; Pires da Silva et al., 2024).

One form of programmatic aging involves costly programs: genetically-determined processes that degrade somatic tissues as by-product of wider, fitness-promoting processes (Gems and Kern, 2024; Gems et al., 2021). In some cases this involves biomass repurposing, where biomass of one tissue is broken down by autophagic processes to release molecular constituents to support functions in another. This occurs to a high degree in semelparous organisms in the process of reproductive death (suicidal reproductive effort), as in many monocarpic plants, and semelparous fish such as Pacific salmon (Gems et al., 2021).

Several lines of evidence support the hypothesis that reproductive death occurs in *C. elegans* hermaphrodites (Gems et al., 2021; Kern et al., 2023). This includes a putative costly program in which intestinal biomass is broken down and repurposed to support production of yolk, that is then vented to support larval growth, leading to intestinal atrophy (a senescent pathology) (Ezcurra et al., 2018; Kern et al., 2021; Sornda et al., 2019). Notably, such biomass repurposing is supported by autophagy, as evidenced by deceleration of intestinal atrophy and yolk pool formation when autophagy is inhibited (Benedetto and Gems, 2019; Ezcurra et al., 2018). Thus, in this particular context autophagy appears to be promoting rather than preventing senescence.

These developments warrant a careful re-examination of the evidence that autophagy is protective against aging in *C. elegans*. In this study we re-examine the question of whether the longevity of *daf-2* insulin/IGF-1 receptor mutants is autophagy dependent. Here the principal form of past evidence involves epistasis tests, where effects on lifespan of reduction of function of genes encoding proteins effecting autophagy is compared in the wild type (N2) and *daf-2* mutants. Details of 8 previous studies involving 45 epistasis experiments are summarised in Table S1. Although it is widely believed that autophagy is essential for *daf-2* mutant longevity (Meléndez et al., 2003), scrutiny of the results of past tests raises doubts about the strength of this claim.

Careful scrutiny of these prior studies identifies six distinct limitations, as follows. (1) A life-shortening effect of inhibition of autophagy does not necessarily indicate its role in the normal aging process, or in *daf-2* mutant longevity. (2) If autophagy is inhibited during development as well as adulthood, a life-shortening effect could result from disruption of normal development. (3) The claim that *daf-2* longevity is autophagy dependent requires evidence that the life-shortening effect in *daf-2* is significantly greater than in wild type, e.g. using Cox proportional hazard analysis, and this is rarely performed. (4) If effects of reducing autophagy are condition dependent, this introduces a potential bias: a risk that investigators might unwittingly select conditions where the data generated supports a role of autophagy in longevity - what may be referred to as *condition selection bias*. Potential determinative conditions that have varied across studies include choice of autophagy-determining gene to inhibit, of *daf-2* mutant allele, and ambient temperature. (5) 5-fluoro-2’-deoxyuridine (FUDR) is sometimes used to facilitate *C. elegans* lifespan assays by preventing progeny hatching; this could potentially affect test outcomes, as shown in other contexts (Aitlhadj and Sturzenbaum, 2010; Anderson et al., 2016; Burnaevskiy et al., 2018; Van Raamsdonk and Hekimi, 2011; Zhao et al., 2019). (6) A final, straightforward issue is whether a given finding proves to be reproducible in subsequent reports under, seemingly, the same conditions.

Of 46 prior tests (Table S1) only 4 present clear evidence that reducing autophagy shortens lifespan more in *daf-2* than in the wild type. Regarding one of these four instances, the effect of *bec-1* RNAi on *daf-2(e1370)* at 15°C (Meléndez et al., 2003), a subsequent study did not replicate this finding (Hars et al., 2007). In two other cases the weaker *daf-2(mu150)* allele was used (Hansen et al., 2008; Patel et al., 2008). In two further studies where large reductions in *daf-2* lifespan were observed (Chang et al., 2017; Minnerly et al., 2017) knockdown of *atg-18* was used. In a 2009 study where effects if knockdown of a wide range of different autophagy genes (14) was tested, adult-limited *atg-18* RNAi was one of only 2/14 that significantly reduced lifespan in the wild type (Hashimoto et al., 2009), suggesting possible *atg-18* idiosyncrasy. The 2009 study includes more than half of all of the prior tests (28/46); strikingly, in only 1/14 genes (*atg-4.1*) did adult-limited RNAi significantly reduce *daf-2(e1370)* lifespan, while for 3/14 genes (*atg-9*, *bec-1* and *unc-51*) it *increased daf-2* lifespan (Hashimoto et al., 2009). One possible reason for the lack of observed life-shortening effects given autophagy knockdown is its use of FUDR at a high concentration.

In this study we assess the condition-dependence of the effects of RNAi knockdown of autophagy genes on *C. elegans* longevity. To this end we have tested the effects of RNAi of six genes in the autophagy pathway on longevity in two different *daf-2* mutants, and a *glp-1* germlineless mutant, at two temperatures. We have also assessed effects of FUDR and prevention of bacterial infection. Our results provide a robust foundation of evidence relating to possible autophagy dependence of *daf-2* and *glp-1* longevity. They suggest a weak and highly-condition dependent contribution of autophagy to *daf-2* and *glp-1* Age.

## Results

### Effects of *atg* RNAi on *daf-2* Age vary with *daf-2* allele

**Figure 1.**
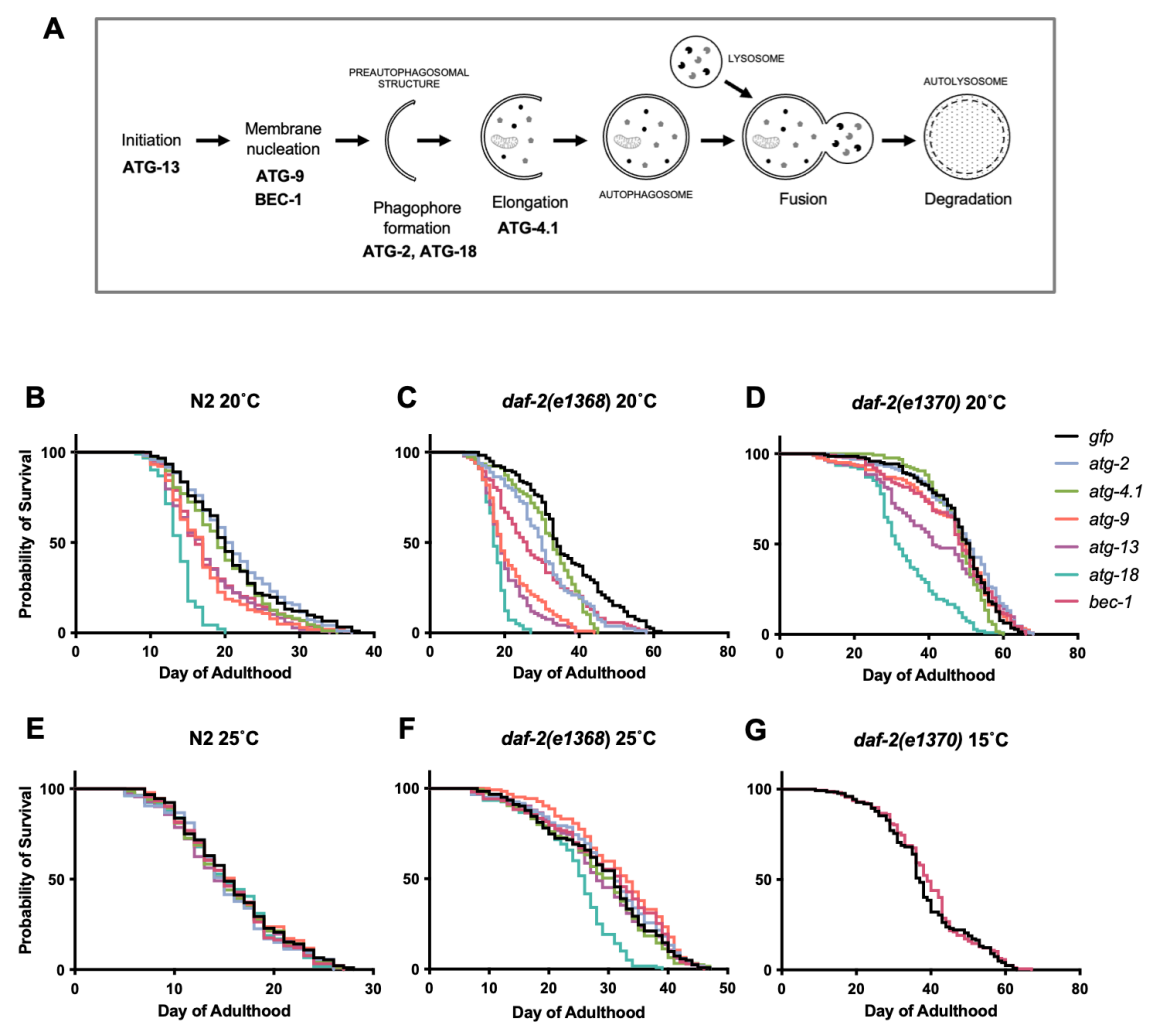
Effects of *atg* RNAi on longevity. (A) The autophagy pathway, and genes tested in this study. (B-D) Effects at 20°C. (B) Effects on N2 (wild type). (C) Effects on *daf-2(e1368)*. (D) Effects on *daf-2(e1370)*. (E, F) Effects at 25°C. (E) Effects on N2. (F) Effects on *daf-2(e1368)*. (G) Effects of *bec-1* RNAi on *daf-2(e1370)* at 15°C. (B-G) summed data, *N* = 2; for individual trials, see Table S2, S3.

We first tested whether effects on lifespan of inhibiting autophagy depend upon *daf-2* allele class. Two *daf-2* mutants were examined. *daf-2(e1368)* is a class 1 (less pleiotropic) mutant where adults show normal behavior at both 20°C and 25°C, while *daf-2(e1370)* is a class 2 (more pleiotropic) mutant where at 25°C adults become paralyzed and cease feeding (Gems et al., 1998). Knockdown of autophagy was performed by RNAi from the L4 (late larval) stage of one of six genes at several stages of the autophagy process: initiation (*atg-13*), membrane nucleation (*atg-9*, *bec-1*), phagophore formation (*atg-2*, *atg-18*) and elongation (*atg-4.1*) (Figure 1A).

**Figure 2.**
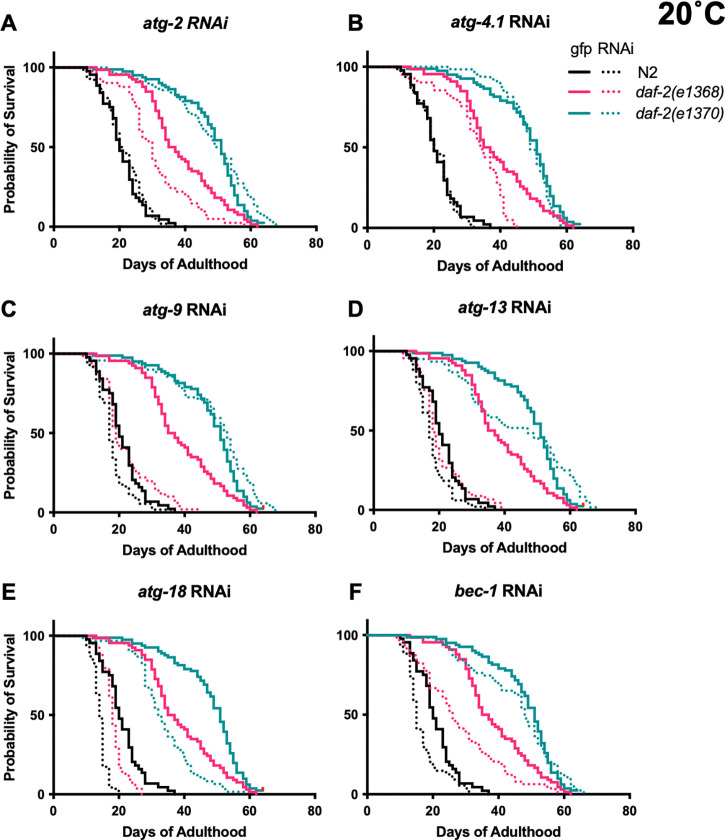
Effects of individual *atg* gene RNAi on the three genotypes (20°C). Summed data, *N* = 2; for individual trials, see Table S2.

Trials were performed at 20°C, and used *gfp* RNAi as a negative control. In N2, statistically significant reductions in lifespan were sometimes seen: *atg-9, atg-13* and, particularly, *atg-18* RNAi shortened lifespan in both trials (summed data, *N* = 2, means: −13.7%, *p* < 0.0001, −12.4%, *p* = 0.0002, −31%, *p* < 0.0001, respectively; log rank test) (Figure 1B, Table S2). For survival curves comparing RNAi effects of individual *atg* genes on the three genotypes, see Figure 2.

RNAi of all six *atg* genes consistently reduced lifespan in the *daf-2(e1368)* mutant, with effects ranging from −10.5% (*p* = 0.0009) for *atg-4.1* to −50.4% (*p* < 0.0001) for *atg-18* (Figure 1C, Table S2). To assess whether *atg* RNAi reduced lifespan more in *daf-2* mutants than in the wild type, Cox proportional hazard (CPH) analysis was used. This showed a significantly greater effect in *daf-2(e1368)* for 4/6 genes (exceptions: *atg-4.1*, *bec-1*) (Table S2). These findings are broadly in line with the earlier observation that *bec-1* and *vps-34* RNAi shortened the lifespan of the *daf-2(mu150)* class 1 mutant but not of N2 at 20°C (Hansen et al., 2008).

To test whether *atg* RNAi is able to fully suppress *daf-2(e1368)* Age, the lifespans of N2 and *daf-2* subjected to *atg* RNAi were compared. Under all six *atg* RNAi conditions, *daf-2(e1368)* still significantly increased mean lifespan, from +15.7% (*p* = 0.0031) for *atg-13* to +60.9% (*p* < 0.0001) for *atg-4.1* (summed data, 20°C, Table S2). This could imply either that *daf-2(e1368)* Age is not fully autophagy dependent, or that it is but autophagy is not fully suppressed by the RNAi treatment.

In *daf-2(e1370)*, *atg* RNAi had far more modest effects on lifespan than in *daf-2(e1368)*. Summed data showed significant reductions in lifespan resulting from RNAi of *atg-9*, *atg-13* and *atg-18* only, with the latter again causing the largest reduction: −8.3% (*p* = 0.012), −17.8% (*p* = 0.0034), and −29.2% (*p* < 0.0001), respectively (Figure 1D, Table S2). However, effects were in no instance significantly greater than in N2 (CPH analysis, Table S2), thus failing to provide evidence that *e1370* longevity is mediated by autophagy.

Taken together, these results suggest that at 20°C autophagy contributes to longevity in class 1 but not class 2 *daf-2* mutants. RNAi effects varied between *atg* genes, with *atg-2* and *atg-4.1* RNAi effects weaker, and *atg-18* RNAi effects generally stronger than the rest.

### Effect of *atg* RNAi on *daf-2* Age is temperature dependent

Results of a previous study performed at 25°C appear to show no greater reduction in lifespan in *daf-2(e1370)* compared to *daf-2(+)* after autophagy knockdown, even when using the *atg-18(gk378)* deletion mutation (Toth et al., 2008) (Table S1), suggesting possible temperature dependence of *atg* RNAi effects. To explore this further, in parallel to tests at 20°C, we also compared effects of *atg* RNAi on lifespan in N2 and *daf-2(e1368)* at 25°C. Effects on *daf-2(e1370)* were not tested, partly because this mutant ceases feeding at 25°C (Gems et al., 1998) which would be expected to interfere with RNAi by feeding.

At 25°C, *atg* RNAi did not shorten N2 lifespan for any of the six genes tested, not even *atg-18* (summed data; Figure 1E, Table S2), i.e. culture at 25°C suppressed the life-shortening effect of *atg* RNAi in N2. Also notable is that the increases in lifespan with *atg-2* and *atg-13* RNAi at 25°C, described in our earlier study (Ezcurra et al., 2018), were not reproduced (discussed below).

At 25°C only *atg-18* RNAi significantly reduced *daf-2(e1368)* mean lifespan, by 15.5% (*p* < 0.0001) (summed data, Figure 1F, Table S2), a reduction that was significantly greater than in N2 (*p* = 0.0012, CPH, summed data only; Table S2). For survival curves comparing RNAi effects of individual *atg* genes on the two genotypes, see Fig. S1. This further underscores the idiosyncrasy of *atg-18*. In one case, *atg-9*, RNAi slightly increased *daf-2* lifespan (+9.8%, *p* = 0.011, summed data).

The initial tests showing that *bec-1* RNAi reduces *daf-2(e1370)* lifespan were performed at 15°C (Meléndez et al., 2003). Taken together with the weaker RNAi effects at 25°C observed here, this suggesting that stronger effects might be seen at 15°C. To explore this we compared effects of *bec-1* RNAi from L4 on N2 and *daf-2(e1370)* at 15°C and 20°C (*N* = 2). However, no suppression of *daf-2(e1370)* Age by *bec-1* RNAi was seen at either temperature (Figure 1G; Table S3), consistent with findings of an earlier study performed at 15°C (Hars et al., 2007) (Table S1).

### FUDR can alter the effect of *atg* RNAi on lifespan

Since the 1980s FUDR, a thymidylate synthase inhibitor and anti-cancer drug, has sometimes been added to *C. elegans* survival trials to prevent progeny production (Gandhi et al., 1980; Mitchell et al., 1979). Notably, two previous reports that observed increases in *C. elegans* lifespan given *atg* RNAi employed FUDR. Our own study saw increases in N2 lifespan after *atg-2* and *atg-13* RNAi from L4 with 15 μM FUDR (Ezcurra et al., 2018). Another study that saw increases in N2 lifespan given adult-limited *atg-7*, *atg-9*, *bec-1* and *unc-51* RNAi used FUDR at a higher concentration, 800 μM (Hashimoto et al., 2009) (E. Nishida, personal communication).

**Figure 3.**
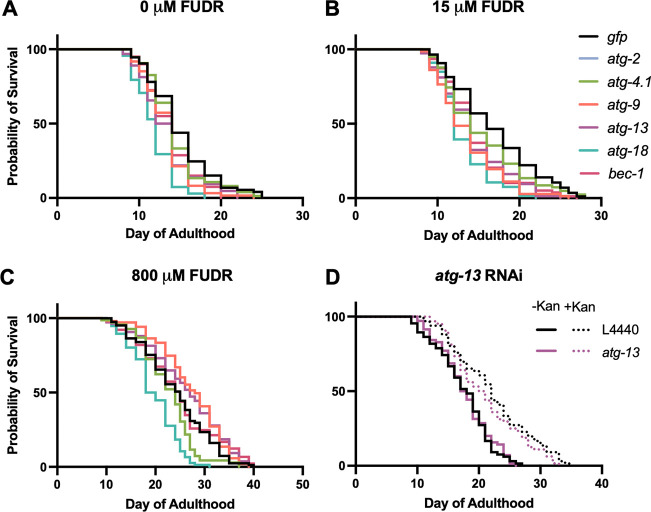
FUDR but not infection alters outcome on *atg* gene RNAi (20°C). (A-C) Effects of 0 μM, 15 μM and 800 μM FUDR. (D) No alteration by kanamycin of *atg-13* RNAi effect on N2 lifespan. Summed data, *N* = 2; for individual trials, see Table S4, S5.

To test for FUDR-dependent effects, we compared the impact of *atg* gene RNAi (*atg-2*, *atg-4.1*, *atg-9*, *atg-13*, *atg-18* and *bec-1*) on N2 lifespan at 20°C with 0, 15 or 800 μM FUDR (*N* = 2). With 0 or 15 μM FUDR, only shortening of lifespan was seen, and FUDR did not significantly alter the effects of RNAi (CPH, Figure 3A, B Table S4).

Addition of 800 μM FUDR increased the lifespan of the *gfp* RNAi negative control by 61.7% (*p* < 0.0001, Table S4), perhaps due to prevention of bacterial proliferation, which can otherwise shorten *C. elegans* lifespan (Garigan et al., 2002; Gems and Riddle, 2000). In the presence of 800 μM FUDR, *atg-9* and *atg-13* RNAi increased lifespan, by +12.5% (*p* = 0.014) and +8.7% (*p* = 0.031), respectively (summed data; Figure 3C, Table S4). Moreover, the life-shortening effect of *bec-1* RNAi, seen with 0 or 15 μM FUDR, was absent. Again, *atg-18* RNAi robustly reduced lifespan under all three conditions (Figure 3C, Table S4). The results using 800 μM FUDR are broadly in line with those of Hashimoto et al. (2009), where *atg-9* and *atg-13* RNAi increased lifespan and *atg-18* was one of only 2/14 genes tested where adult-limited RNAi decreased N2 lifespan. This suggests that the increases in lifespan after *atg* RNAi reported in that study could have reflected its use of 800 μM FUDR (Hashimoto et al., 2009).

### *atg* RNAi shortens lifespan in the absence of bacterial infection

Under standard laboratory culture conditions, *C. elegans* lifespan is limited by infection by the *E. coli* food source, such that prevention of bacterial proliferation substantially increases lifespan (Garigan et al., 2002; Gems and Riddle, 2000). The preceding results could imply that 800 μM FUDR suppresses life-shortening effects of *atg* RNAi by preventing bacterial infection. Xenophagy is generally protective against infection in *C. elegans* (Balla et al., 2019; Jia et al., 2009). Thus, reduction in lifespan given *atg* gene knockdown could reflect increased susceptibility to infection.

To probe this hypothesis, we compared effects on N2 lifespan of *atg-13* RNAi at 20°C in the absence or presence of the antibiotic kanamycin (Kan), to suppress bacterial infection. In the absence of Kan, *atg-13* RNAi caused a slight reduction in lifespan in these trials that did not reach statistical significance (Figure 3D, Table S5), in contrast to other trials in this study (Table S2, S4). As expected, application of Kan extended *C. elegans* lifespan (+27.1%, *p* < 0.0001, summed data, Table S5), and in its presence *atg-13* RNAi resulted in the same slight reduction in lifespan (Figure 3D, Table S5). These results suggest that life-shortening effects of *atg* RNAi are not solely attributable to increased susceptibility to *E. coli* infection. Moreover, they do not indicate marked condition dependency in *atg* RNAi effects on lifespan with respect to *E. coli* proliferative status.

### *atg-2* and *atg-18* RNAi robustly suppress *glp-1(e2141)* Age

We next explored more widely the reproducibility and condition dependence of the requirement for autophagy of *C. elegans* longevity. Prevention of germline development by laser surgery or mutation increases lifespan in *C. elegans* hermaphrodites (Arantes-Oliveira et al., 2002; Hsin and Kenyon, 1999; Pires da Silva et al., 2024). Prior tests for possible autophagy dependence of such longevity have largely used the temperature-sensitive *glp-1(e2141)* germline proliferation mutant, which is fertile at 15°C but sterile and with greatly reduced germline development at 25°C. A key study reported strong and reproducible suppression of *glp-1* Age by RNAi of five autophagy-related genes, including *atg-18* and *bec-1* (Lapierre et al., 2011) (previous findings summarized in Table S6).

**Figure 4.**
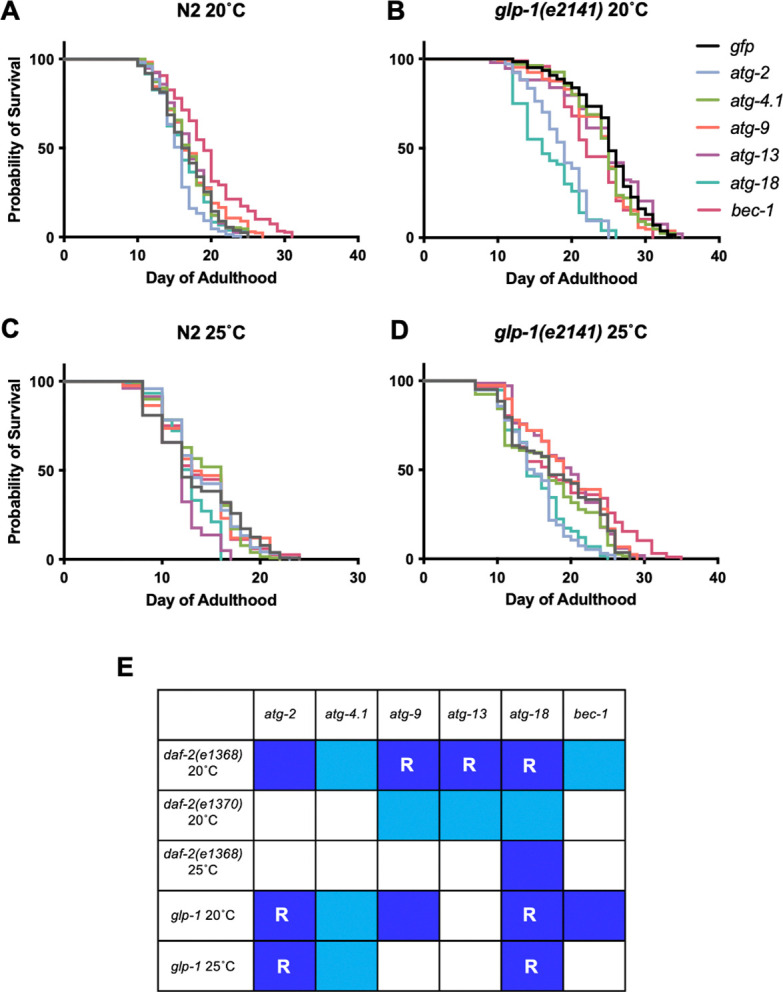
Effects of *atg* RNAi on *glp-1(e2141)* lifespan. (A, B) 20°C. (A) Effects on N2. (B) Effects on *glp-1*. (C, D) 25°C. (C) Effects on N2. (D) Effects on *glp-1*. Summed data, *N* = 2–5; for individual trials, see Table S7. (E) Overview of effects of suppression of Age by RNAi of genes specifying autophagy. Dark blue, reduction of *daf-2* or *glp-1* Age that is significantly greater than in N2. Light blue, reduction of *daf-2* or *glp-1* Age that is not significantly greater than in N2. R, robust suppression, i.e. Age is largely suppressed.

**Figure 5.**
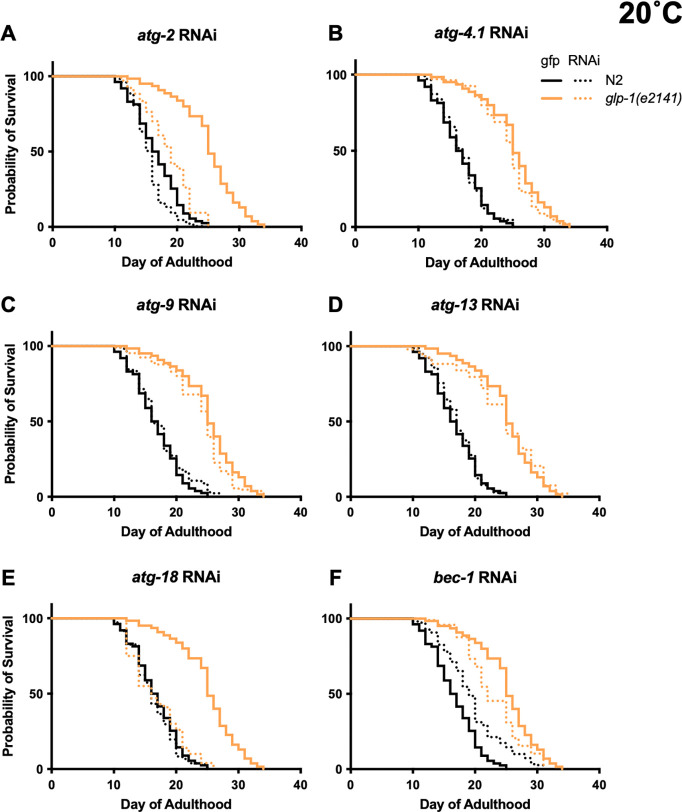
Effects of individual *atg* gene RNAi on N2 and *glp-1(e2141)* lifespan, 20°C. Summed data, *N* = 2–5; for individual trials, see Table S7.

We first tested the effect of *atg* RNAi on *glp-1* longevity with animals raised from L1 until L4 stage at 25°C, and maintained at 20°C thereafter, similar to previous studies (Table S6). Again, effects of *atg-2*, *atg-4.1, atg-9, atg-13*, *atg-18* and *bec-1* RNA were tested. In the RNAi control *glp-1* increased mean lifespan by +50.9% (*p* < 0.0001, summed data, Table S7). *glp-1* lifespan was significantly reduced by *atg-2*, *atg-4.1*, *atg-9*, *atg-18* and *bec-1* RNAi (but not *atg-13* RNAi), and effects were greater in *glp-1* than N2 in all cases except *atg-4.1* (Figure 4A, B, Figure 5, Table S7). However, suppression was only robust (of a large magnitude) for *atg-2* and *atg-18* RNAi (Figure 4B, Figure 5). Under all six *atg* RNAi conditions, *glp-1* still significantly increased mean lifespan, from +4.12% (*p* = 0.0003) for *atg-18* to +43.8% (*p* < 0.0001) for *atg-4.1* (summed data, 20°C, Table S7). This could imply either that *glp-1* Age is not fully autophagy dependent, or that it is but autophagy is not fully suppressed by the RNAi treatment.

In tests with *daf-2(e1368)* life-shortening effects of *atg* RNAi were largely absent at 25°C (apart from *atg-18*) (Figure 1F, Table S2). We therefore wondered whether temperature might also influence the outcome of *atg* RNAi treatment in *glp-1* mutants. To test this we examined RNAi effects on lifespan at 25°C. Under these conditions, *glp-1* lifespan was significantly reduced by only *atg-2, atg-4.1*, and *atg-18* RNAi (Figure 4C, D, Fig. S2, Table S7), and effects were significantly greater in *glp-1* than N2 only with *atg-2* and *atg-18* RNAi (CPH analysis, Table S7). In summary, of the six genes tested only *atg-2* and *atg-18* RNAi robustly suppressed *glp-1* Age, and RNAi effects were weaker at 25°C.

For an overview of the effects of RNAi on *daf-2* and *glp-1*, see Figure 4E. In 11 of the 30 conditions tested RNAi knockdown of autophagy caused a greater reduction in lifespan in the long-lived mutant. In 7/30 the RNAi effect was robust, i.e. the mutant longevity was largely suppressed. This suppression was highly condition dependent, differing according to gene knocked down, temperature, *daf-2* allele used, and between *daf-2* and *glp-1*. Notably, *atg-18* RNAi robustly suppressed longevity in both *daf-2* and *glp-1*, whereas *atg-2* RNAi did so only in *glp-1* mutants.

### Why are effects of *atg-18* RNAi stronger than those of other *atg* genes?

The unusually strong life-shortening effects of *atg-18* RNAi could imply the presence of pleiotropic effects not directly related to autophagy. Given that longevity due to either *daf-2* mutation or germline loss are wholly dependent on the FOXO transcription factor DAF-16 (Hsin and Kenyon, 1999; Kenyon et al., 1993), we wondered whether *atg-18* RNAi might inhibit DAF-16. To probe this two approaches were taken. First, we used a constitutive dauer formation assay. High population density and food depletion causes *C. elegans* larvae to form developmentally arrested dauer larvae (Cassada and Russell, 1975). *daf-2* mutants undergo constitutive dauer arrest (the Daf-c phenotype) in a temperature-sensitive manner, and this is fully suppressed by *daf-16*(−) (Riddle et al., 1981). However, using a sensitive assay (*daf-2(m41)*, 22°C), we detected no reduction in the number of dauers formed given *atg* gene RNAi (*atg-2*, *atg-13* and *atg-18*) (Fig. S3A).

Second, we tested a GFP reporter for a gene whose expression is elevated in *daf-2* mutants in a *daf-16*-dependent manner (*ftn-1*) (Ackerman and Gems, 2012). GFP levels were compared in *daf-2(m577)* and *daf-16(mgDf50)*; *daf-2* backgrounds. As expected, GFP levels were higher in *daf-2* than in *daf-16; daf-2* (Fig. S4). RNAi did not suppress the *daf-2*-induced increase of *ftn-1::gfp* expression (Fig. S3B). These findings argue against a pleiotropic effect of *atg-18* on DAF-16 function.

### Inhibiting autophagy does not reduce vitellogenin accumulation

Finally, we further investigated the hypothesis that autophagy promotes biomass repurposing in *C. elegans*. Inhibition of yolk synthesis or of autophagy delays intestinal atrophy and yolk pool accumulation, suggesting that intestinal biomass is repurposed for yolk synthesis (Benedetto and Gems, 2019; Ezcurra et al., 2018; Sornda et al., 2019). In principle, this could involve repurposing into yolk protein (vitellogenin) or yolk lipid. To test the former possibility, wild-type hermaphrodites or *fog-2(q71)* (feminization of germline) mutant females were subjected to *atg* RNAi. The *fog-2* mutant, which lacks self-sperm and so lays no eggs, was used to avoid possible effects of *atg* RNAi on fertility, reduction of which can increase vitellogenin levels within nematodes (Sornda et al., 2019).

**Figure 6.**
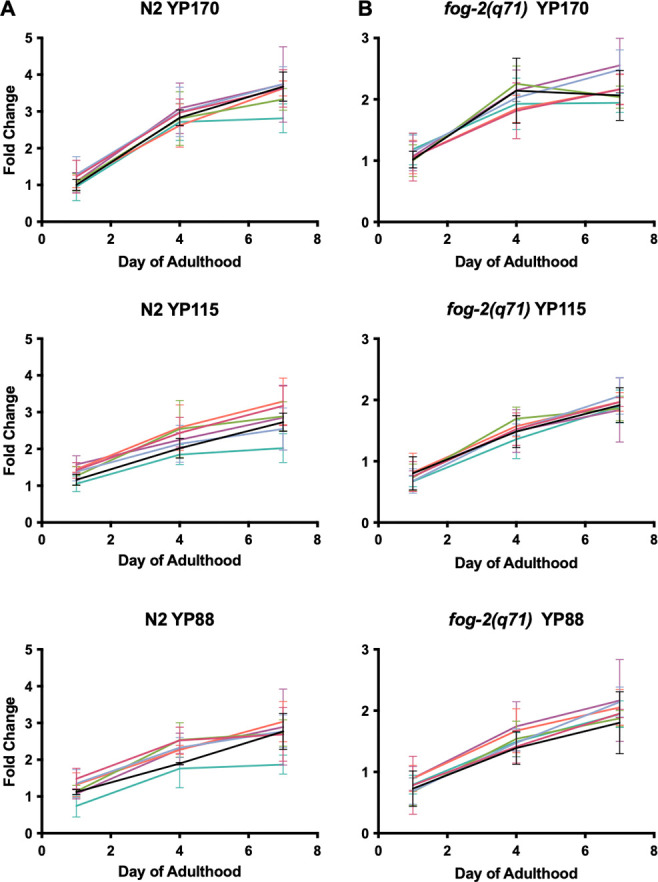
Effects of *atg* RNAi on vitellogenin accumulation. Fold change of yolk proteins YP170, YP115 and YP88 in N2 and *fog-2* normalized to *gfp* day 1 (*N* = 3). In no case is vitellogenin level significantly different to that in the *gfp* RNAi control at any time point (two-way ANOVA, Table S8). For raw data for vitellogenin levels see Supplementary Dataset 2.

For none of the six *atg* genes tested did RNAi detectably reduce yolk protein levels, either in N2 hermaphrodites or *fog-2* females (Figure 6). This implies that autophagic machinery, including that in the intestine, does not enhance yolk protein production. Thus, if intestinal biomass repurposing occurs, then it likely supports yolk secretion or yolk lipid production.

## Discussion

Overall, the effects of reducing *atg* gene function described here are ambiguous with respect to the role of autophagy in *daf-2* or *glp-1* longevity, neither clearly supporting or excluding it. However, they are consistent with a role of autophagy in the longevity of weaker, class 1 *daf-2* alleles at lower temperatures (Table S2). Class 1 allele-limited suppression of *daf-2* Age has been seen previously, for example in epistasis tests with *daf-12* (encoding a dafachronic acid receptor) (Gems et al., 1998; Larsen et al., 1995) and *skn-1* (Nrf2-like transcription factor) (Tullet et al., 2008). This could reflect a role of autophagy in longevity assurance limited to conditions of mild IIS reduction, or sensitivity to differential effects of *daf-2* on distinct downstream signaling outputs, such as phosphatidylinositol 3-kinase and Ras signaling (Patel et al., 2008).

*atg* RNAi suppresses *daf-2(e1368)* Age at 20°C but not 25°C (Figure 1B,E, Table S2). Given that for hypomorphic *daf-2* alleles (such as *e1368* and *e1370*) many mutant traits, including Age, show some degree of temperature sensitivity (Gems et al., 1998; Riddle and Albert, 1997), the absence of suppression at 25°C may reflect the increased severity of the *daf-2(e1368)* mutant phenotype at this higher temperature. However, life-shortening effects of *atg* RNAi on N2 are also reduced at 25°C, suggesting that additional mechanisms may also mediate such temperature sensitivity.

### Resolving discrepancies between past studies of autophagy dependence of *daf-2* Age

The increasing non-reproducibility of many experimental findings, that has been referred to as the *reproducibility crisis*, is a problem that particularly afflicts biological and biomedical research (Lithgow et al., 2017; Prinz et al., 2011; Ritchie, 2020; Voelkl et al., 2020). A strength of *C. elegans* as an experimental model is the relative ease with which such discrepancies can be resolved, at least in principle. This is thanks to the use of standardized culture conditions across the *C. elegans* research community, and of nematode strains based on the same isogenic wild-type strain (N2), plus the relatively low cost and short duration of experiments.

Regarding lifespan assays in particular, possible reasons for discrepant findings include clearly identifiable differences in experimental design, such as use or not of FUDR. Less obvious causes include cryptic variation in genetic background (Zhao et al., 2019), or subtle differences in culture conditions (e.g. due to batch variation in Bacto Peptone) (Petrascheck, 2014).

Our findings potentially resolve several discrepancies between earlier studies relating to the possible role of autophagy in *daf-2* mutant longevity. Previous studies found that inhibiting autophagy either suppressed or enhanced *daf-2* Age, or had no effect (Table S1). One study showing suppression used the weak class 1 allele *daf-2(mu150)* (Hansen et al., 2008). This is consistent with our observation that, *atg-18* aside, *atg* RNAi can suppress Age in a class 1 but not a class 2 allele (Figure 1C, D Table S2).

Does *bec-1* RNAi suppress *daf-2(e1370)* Age? The initial test suggesting this was performed at 15°C, and employed RNAi by injection of the mothers of assayed individuals (Meléndez et al., 2003). A subsequent study at 15°C, where both mothers and adult progeny were exposed to *bec-1* RNAi by feeding appears not to shorten *daf-2(e1370)* lifespan (Hars et al., 2007). Several further trials under different conditions did not observe a life-shortening effect either (Hashimoto et al., 2009; Toth et al., 2008). In the present study, we saw no shortening of *daf-2(e1370)* lifespan in summed data with adult-limited *bec-1* RNAi by feeding, at either 15°C or 20°C (Figure 1C, F, Fig. S2, Table S2, S3). Taken together with earlier evidence, our observations suggest that suppression of *daf-2(e1370)* Age by *bec-1* RNAi is not a readily reproducible finding.

Several studies reported lifespan extension following *atg* RNAi (Ezcurra et al., 2018; Hashimoto et al., 2009). Here we were able to reproduce this effect for several *atg* genes by applying high dose FUDR (800 μM), thus recapitulating findings by Hashimoto et al (2009), and potentially accounting for the life span increases seen in that study; it was only subsequent to that study that evidence emerged of the capacity of FUDR to alter effects of interventions that impact lifespan (Aitlhadj and Sturzenbaum, 2010; Anderson et al., 2016; Burnaevskiy et al., 2018; Van Raamsdonk and Hekimi, 2011; Zhao et al., 2019).

Regarding issues with replicating published findings, in an earlier study we observed increases in N2 lifespan given *atg-2* and *atg-13* RNAi (Ezcurra et al., 2018). However, this finding proved not to be robust to replication, either at 20°C (Figure 1B, 2A,D, 3A,B, 4A, 5A,D, Table S2, S4, S7) or 25°C (Figure 1E, 4C, Fig. S1A,D, S2A,D, Table S2, S7), for reasons that remain unclear. This again illustrates the value, as with the effects of *bec-1* RNAi on *daf-2(e1370)* Age, of repeated verification of effects of interventions on *C. elegans* lifespan.

As with *daf-2*, our tests with the *glp-1* germline proliferation mutant did not clearly support the view that mutant longevity is autophagy dependent. However, consistent with an earlier study (Lapierre et al., 2011), we observed that *atg-18* and *bec-1* RNAi reduce *glp-1* lifespan more than in wild-type at 20°C.

### Why does *atg-18* RNAi more strongly suppress *daf-2* Age?

Of the six autophagy-determining genes tested here, RNAi of *atg-18* showed a greater capacity to suppress both *daf-2* and *glp-1* Age than the other five. This is consistent with an earlier finding that *atg-18* RNAi suppresses *eat-2* Age more strongly than does *lgg-1*/*lgg-2* double RNAi (Gelino et al., 2016). *atg-18* appears to have replaced *bec-1* as the gene of choice for autophagy-related epistasis studies in *C. elegans* (Chang et al., 2017; Minnerly et al., 2017).

Notably, *atg-18* was one of only 2/14 autophagy genes tested where RNAi during development caused a high level (>50%) of larval growth arrest or lethality (Hashimoto et al., 2009). The idiosyncratic effects of *atg-18* RNAi could reflect either greater inhibition of autophagy, or pleiotropy in which processes other than autophagy are altered. Regarding pleiotropy, several proteins in the canonical autophagy pathway have recently been found to participate in other processes. For example, ATG8 (LGG-1 in *C. elegans*) functions in trafficking of single-membrane organelles (Nieto-Torres et al., 2021), while *C. elegans* ATG-16.2 contributes to neuronal exopher formation via its WD40 domain (Yang et al., 2024). In an as yet unexplained instance of *atg* gene idiosyncrasy, mutation of *atg-16.2*, *atg-18* and *bec-1* retards the cell cycle in *C. elegans* germline cells, while that of *atg-7* does not (Ames et al., 2017).

Concerning possible *atg-18* pleiotropy specifically, prior work provides several clues. ATG-18 is a predicted WIPI (WD repeat protein interacting with phosphoinositides) family member. WIPI proteins contain a WD40 repeat region that binds to phosphatidylinositol 3-phosphate (PtdIns(3)P) in lipid membranes, which mediates their function in vesicle trafficking (Grimmel et al., 2015). Their best known role is in autophagosome generation, where PtdIns(3)P formation at the endoplasmic reticulum provides an initiation signal, to which the WIPI protein binds and recruits other autophagy pathway proteins (Grimmel et al., 2015). However, WIPI proteins have diverse functions beyond autophagy, that vary across taxa. Studies from the budding yeast *Saccharomyces cerevisiae* and the slime mold *Dictyostelium discoideum* have identified roles of WIPI proteins in several additional steps in autophagy, including autophagosome lipidation, elongation, closure and fusion with lysosomes (Vincent et al., 2021). If *atg-18* contributes to several steps in autophagosome formation and trafficking in *C. elegans*, this might explain why its RNAi results in particularly strong inhibition of autophagy.

At the same time, such functional versatility in WIPI proteins is also consistent with pleiotropy, as are several other observations. In humans there are four WIPI proteins, WIPI1 - WIPI4; *C. elegans atg-18* is more closely related to WIPI1/WIPI2, while *epg-6* more closely resembles WIPI3/WIPI4 (Lu et al., 2011). Notably, deletion mutations of *atg-18* and *epg-6* appear to cause similar reductions in levels of autophagy, yet only *atg-18* shortens lifespan (Takacs et al., 2019). In yeast, WIPI1 was initially characterized as a trans-Golgi/endosomal protein involved in recycling between the vacuole and Golgi (Jeffries et al., 2004), further supporting potential non-autophagic roles.

Of particular relevance here, a *C. elegans* study of rescue of longevity in *daf-2(e1370); atg-18(gk378)* double mutants by tissue-specific expression of *atg-18*(+) revealed a major role of *atg-18* in food-sensing chemosensory neurons, potentially reflecting a vesicle trafficking role in neurosecretion (possibly of neuropeptides) (Minnerly et al., 2017). Whether such a role is unique to *atg-18*, or shared by other *atg* genes remains unclear; however, the canonical autophagy pathway does play a role in development of certain neurons in *C. elegans* (Stavoe et al., 2016), and in mammals post-developmentally in synaptic vesicle trafficking (Binotti et al., 2015; Hernandez et al., 2012) and degradation of postsynaptic receptors (Rowland et al., 2006).

In conclusion, the possibility remains that the more severe effects of *atg-18* RNAi reflect pleiotropic effects unrelated to autophagy. More generally, results of the use of *atg* gene knockdown to test for autophagy dependence in biological processes should be interpreted with caution.

### Autophagy in biomass repurposing during programmatic aging

Autophagic processes play a major role in tissue degeneration related to biomass repurposing in semelparous organisms (that reproduce once and then die), particularly plants (Avila-Ospina et al., 2014; Gems et al., 2021). Previous studies support the hypothesis that intestinal biomass is repurposed for synthesis of yolk that, subsequent to egg laying, is vented to support larval development (Kern et al., 2021), and that autophagy facilitates this biomass conversion (Ezcurra et al., 2018; Sornda et al., 2019). If late-life mortality is promoted by intestinal atrophy, then preventing it should extend lifespan. Consistent with this, intestinal atrophy and yolk production are suppressed in *daf-2* mutants (Depina et al., 2011; Ezcurra et al., 2018), and blocking yolk production both retards intestinal atrophy and modestly increases lifespan (Ezcurra et al., 2018; Murphy et al., 2003; Sornda et al., 2019). This could imply that inhibiting autophagy, by retarding intestinal atrophy could extend lifespan under some conditions, and several earlier studies observed life extension after *atg* RNAi (Ezcurra et al., 2018; Hashimoto et al., 2009; Wilhelm et al., 2017).

The results of the present study are in line with the view that intestinal atrophy, though a salient feature of *C. elegans* senescence, by itself contributes only weakly to late-life mortality. Consistent with this, inhibition of *daf-2* using auxin-induced degradation of DAF-2 protein can strongly increasing lifespan even when initiate only at advanced ages, long after intestinal atrophy has occurred, and without any detectable reversal of major senescent pathologies (Molière et al., 2024; Venz et al., 2021). One possibility is that *atg* RNAi has antagonistic effects on late-life mortality: modestly reducing it due to suppression of intestinal atrophy (as seen with when vitellogenin synthesis is inhibited) (Ezcurra et al., 2018; Murphy et al., 2003; Sornda et al., 2019), but also increasing it, due to disruption of essential cellular functions.

The gut-to-yolk biomass repurposing hypothesis drew particularly on the observation that blocking yolk protein (vitellogenin) synthesis or autophagy reduced intestinal atrophy and pseudocoelomic yolk pool size (Ezcurra et al., 2018; Sornda et al., 2019); given that yolk pools contain yolk protein and lipid (Ezcurra et al., 2018; Garigan et al., 2002; Herndon et al., 2002; Palikaras et al., 2017), such putative biomass repurposing could increase production of yolk protein, yolk lipid or both. Here we tested whether *atg* RNAi reduces yolk protein levels, and found that it did not (Figure 6). If the hypothesis is correct, then this would support the view that promotion by autophagy of repurposing of intestinal biomass into yolk involves yolk lipid rather than yolk protein, or overall yolk secretion. In principle, this could involve mass export of stored lipid, either by means of lipophagy, or perhaps secretory autophagy (Ponpuak et al., 2015).

### Concluding remarks

This study reassesses the claim that *daf-2* Age is autophagy dependent. While the results do not entirely prove or disprove this claim, they clearly show that evidence of dependence varies greatly with context, and suggest that autophagy only contributes substantially to *daf-2* Age given a weak reduction in IIS.

One limitation in establishing the role of autophagy in *daf-2* mutant longevity is the difficulty of reliably measuring its level of activity. While several assays using fluorescent reporters to identify putative autophagosomes suggest possible increases in autophagy levels (Chang et al., 2017; Hansen et al., 2008; Meléndez et al., 2003), *daf-2* mutants also show marked reductions in both protein synthesis (Depuydt et al., 2013) and protein turnover (Dhondt et al., 2016; Visscher et al., 2016), more consistent with a reduction rather than an increase in autophagy, and in line with observed suppression of intestinal atrophy in *daf-2* mutants (Ezcurra et al., 2018).

One issue raised by condition dependence of a given effect is that it creates a risk of condition selection bias, where choice of experimental conditions can bias the outcome of an experiment. Where condition dependence has been identified, condition selection bias may be avoided by selection of multiple conditions to test a given hypothesis. In the present case, this could entail comparing effects on *daf-2(e1368)* and *daf-2(e1370)* at 20°C, ideally without FUDR use, and RNAi tests with multiple *atg* genes, at least until the reason for the idiosyncratic effects of *atg-18* RNAi are understood. General questions relating to how to deal with condition dependency have been discussed previously (Munafò and Davey Smith, 2018; Voelkl et al., 2020).

A second, wider issue is that condition dependence risks generating a situation where different research articles make conflicting claims, supporting different views held by different groups of researchers. (For example, the thorough study by Hashimoto et al (2009) is rarely cited). This can obstruct scientific progress, and runs contrary to very nature of scientific research. In the politics of populism and autocracy, realities are often specified by those in power (Arendt, 1951; Orwell, 1949), such that it is possible to speak of “alternative facts”, as in the notorious remark once made to journalists by a counsellor to a US president (Bradner, 2017). Such a “you have your facts and we have ours” position is not consistent with the scientific endeavor.

For science to progress effectively it is necessary for research communities to resolve discrepancies between published findings, though the work required can be tedious, a task akin to washing the dishes in a communal household. It requires identifying and flagging findings that are either condition dependent or, seemingly, unreproducible and, where possible, distinguishing the two. For *C. elegans* at least, such a tidying process is highly feasible, which is a particular virtue of this model organism.

## Materials and Methods

### Culture methods and strains

*C. elegans* maintenance was performed using standard protocols (Brenner, 1974). Unless otherwise stated, all strains were on nematode growth media (NGM, containing Bacto Peptone) with plates seeded with *E. coli* OP50 to provide a food source. An N2 hermaphrodite stock recently obtained from the Caenorhabditis Genetics Center was used as wild type (N2H) (Zhao et al., 2019). Genotypes of most mutants used are as described in Wormbase (www.wormbase.org). Strains used included CB4027 *glp-1(e2141)*, GA633 *daf-2(m577)*; *wuIs177 [Pftn-1::gfp lin-15(+)]*, GA643 *daf-16(mgDf50)*; *daf-2(m577)*; *wuIs177 [Pftn-1::gfp lin-15(+)]*], GA1930 *daf-2(e1370)*, GA1945 *daf-2(m41)*, GA1960 *daf-2(e1368)*, and JK574 *fog-2(q71) V*.

### Daf-c assay

*daf-2(m41)* worms were maintained for at least two generations on the RNAi feeding strains prior for the Daf-c assay. Mothers were transferred onto RNAi plates at L4 and incubated at 22°C for 166 hr, and then progeny (dauers and non dauers) were scored.

### Epifluorescence microscopy

Nematodes were anaesthetized with 10 μl 2 mM levamisole on 2% agar pads prior to imaging. For imaging, we used either a Zeiss Axioskop 2 plus microscope with Hamamatsu ORCA-ER digital camera C4742–95 and Volocity 6.3 software (Macintosh version) for image acquisition; or an ApoTome.2 Zeiss microscope with a Hamamatsu digital camera C13440 ORCA-Flash4.0 V3 and Zen software for image acquisition.

### RNA-mediated interference (RNAi)

RNAi by feeding was performed as described previously (Kamath et al., 2001) using plasmids transformed into *E. coli* OP50(*xu363*) (Xiao et al., 2015) unless otherwise stated. RNAi was initiated from the L4 stage, unless otherwise stated. Inserts of all RNAi feeding clones were confirmed by sequencing. Origins of plasmids in RNAi feeding strains: *atg-13*, *bec-1*: Ahringer library (Kamath et al., 2003); *atg-2*, *atg-4.1*, *atg-9*: Vidal library (Rual et al., 2004). A new *atg-18* RNAi plasmid was prepared using primers gcctccacttcctgttgaag and gagactcttttcgtcggca, and plasmid L4440.

### Survival analysis

Nematodes were maintained at a density of 25–30 per plate, and transferred daily during the egg laying period, and every 6–7 days thereafter. The L4 stage was defined as day 0. Mortality was scored every 1–2 days, with worms scored as alive if they showed any movement, either spontaneously or in response to gentle touch with a worm pick.

### Electrophoresis of *C. elegans* yolk protein

N2 and *fog-2(q71)* worms were synchronized by performing an egg lay and allowing nematodes to grow at 20°C until they reached the L4 stage. L4 worms were then transferred to fresh seeded RNAi plates and maintained under standard culture conditions at 20°C. Five worms were harvested at day 1, day 4 and day 7 into microcentrifuge Eppendorf tubes containing 10 μl M9 buffer. Before running the gel, 10 μl of 2x Laemmli sample buffer was added. Samples were incubated at 70°C and vortexed periodically for 15 min. Samples were then incubated at 95°C and vortexed periodically for 5 min, and were centrifuged at 6,000 rpm for 15 min.

10 μl of sample was loaded into wells of Invitrogen NuPAGE Bis-Tris protein gels. 5 μl of CozyHi prestained protein ladder was loaded at the left side of the gels. The running buffer used was 5% XT MOPS (Bio-rad). The gels were then run at 150V for 2 hr. Gels were removed from cassette and placed in 100 ml of Coomassie staining solution overnight. Gels were then washed with distilled water three times, then placed into destaining solution and soaked for 40 min. Gels were then washed with distilled water and stored at 4°C until imaged. Gels were imaged and saved as 8 bit grayscale TIF files. Images of gels were analysed using Fiji software. Bands of interest (e.g. myosin, YP170, YP115, YP88) were selected manually based on their molecular weight, and intensity of the bands was measured and exported to Microsoft Excel for further analysis.

### Statistical Analysis

Statistical tests were performed on raw data using either Prism 9.0 (GraphPad Software, Boston, MA, USA) or JMP Pro 15 (JMP Statistical Discovery LLC, Cary, NC, USA) unless otherwise stated. The specific tests and post hoc corrections performed are described in the figure legends. To detect alterations in lifespan, the log rank test was used. To compare the magnitude of changes in lifespan, Cox Proportional Hazard (CPH) analysis was used. To compare yolk protein levels, a two-way ANOVA was used. For lifespan trials, no statistical methods were used to predetermine sample size. The experiments were not randomized. The investigators were not blinded to allocation during experiments and outcome assessment.

## Supplementary Material

Supplement 1

Supplement 2
