# Supplementary material for: Condition-dependent effects of knockdown of autophagy on C. elegans longevity
Source: bioRxiv. 2025 Jul 31:2025.07.28.667102. Preprint. [Version 1] doi: 10.1101/2025.07.28.667102 (PMC12324348; doi:10.1101/2025.07.28.667102)
Supplement: Supplement 2 [file NIHPP2025.07.28.667102v1-supplement-2.pdf]

bioRxiv preprint doi: https://doi.org/10.1101/2025.07.28.667102; this version posted July 31, 2025. The copyright holder for this preprint (which was not certified by peer review) is the author/funder, who has granted bioRxiv a license to display the preprint in perpetuity. It is made available under aCC-BY 4.0 International license.

Supplementary Information

Condition-dependent effects of knockdown of autophagy on *C. elegans* longevity

Contents Summary

Supplementary Figure 1. Effects of individual *atg* gene RNAi on N2 and *daf-2(e1368),* 25˚C (summed data).

Supplementary Figure 2. Effects of individual *atg* gene RNAi on N2 and *glp-1(e2141)* lifespan, 25˚C (summed data).

Supplementary Figure 3. Tests for effects of *atg* RNAi on DAF-16 function.

Supplementary Table 1. Previous reports of effects of inhibition of autophagy on *daf-2* Age.

Supplementary Table 6. Previous reports of effects of inhibition of autophagy on GSC(-) Age.

bioRxiv preprint doi: https://doi.org/10.1101/2025.07.28.667102; this version posted July 31, 2025. The copyright holder for this preprint (which was not certified by peer review) is the author/funder, who has granted bioRxiv a license to display the preprint in perpetuity. It is made available under aCC-BY 4.0 International license.

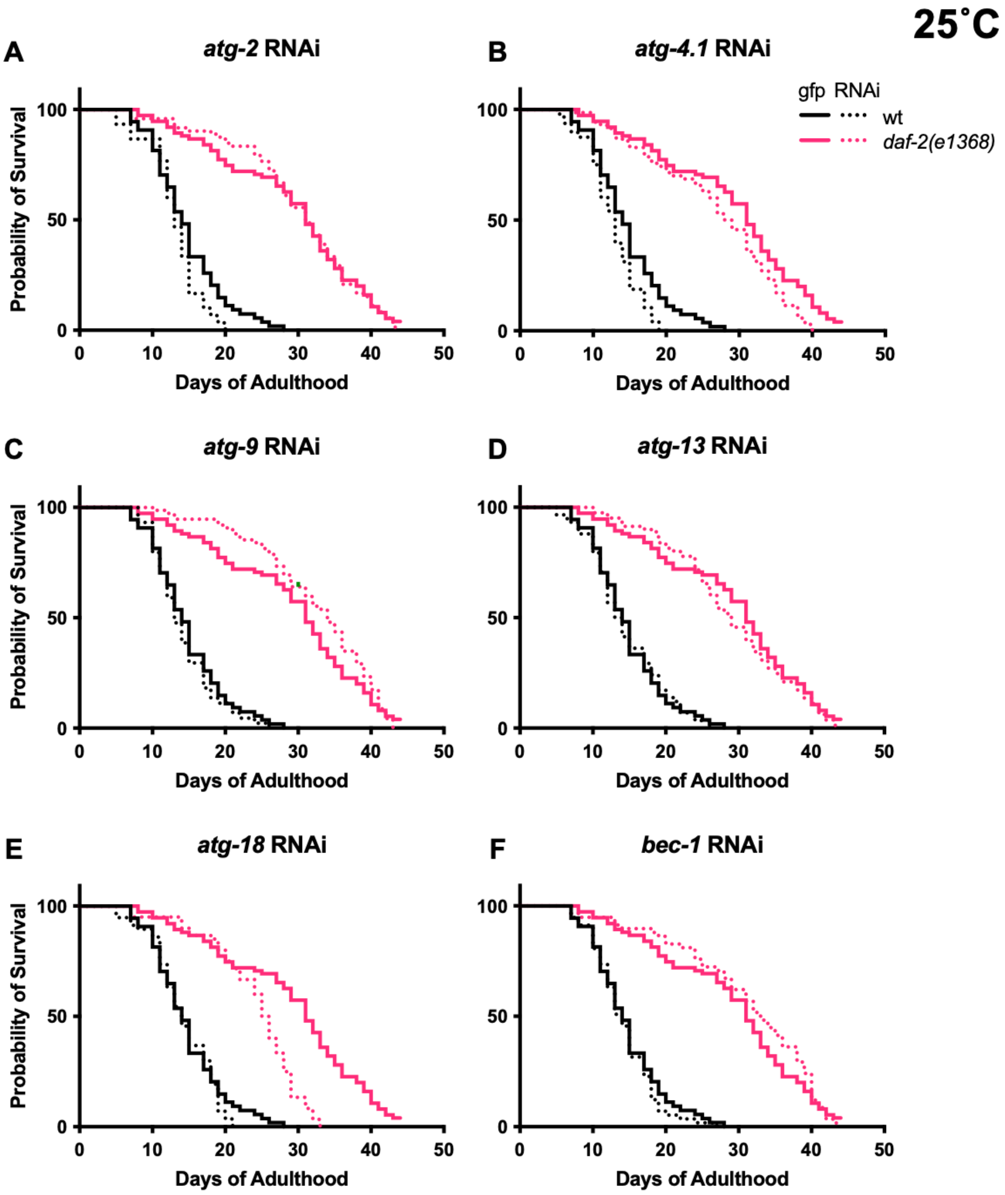

Supplementary Fig. 1. Effects of individual *atg* gene RNAi on N2 (wild type) and *daf-2(e1368)* lifespan (25˚C). Summed data, $N = 2$; for individual trials, see Table S2.

bioRxiv preprint doi: https://doi.org/10.1101/2025.07.28.667102; this version posted July 31, 2025. The copyright holder for this preprint (which was not certified by peer review) is the author/funder, who has granted bioRxiv a license to display the preprint in perpetuity. It is made available under aCC-BY 4.0 International license.

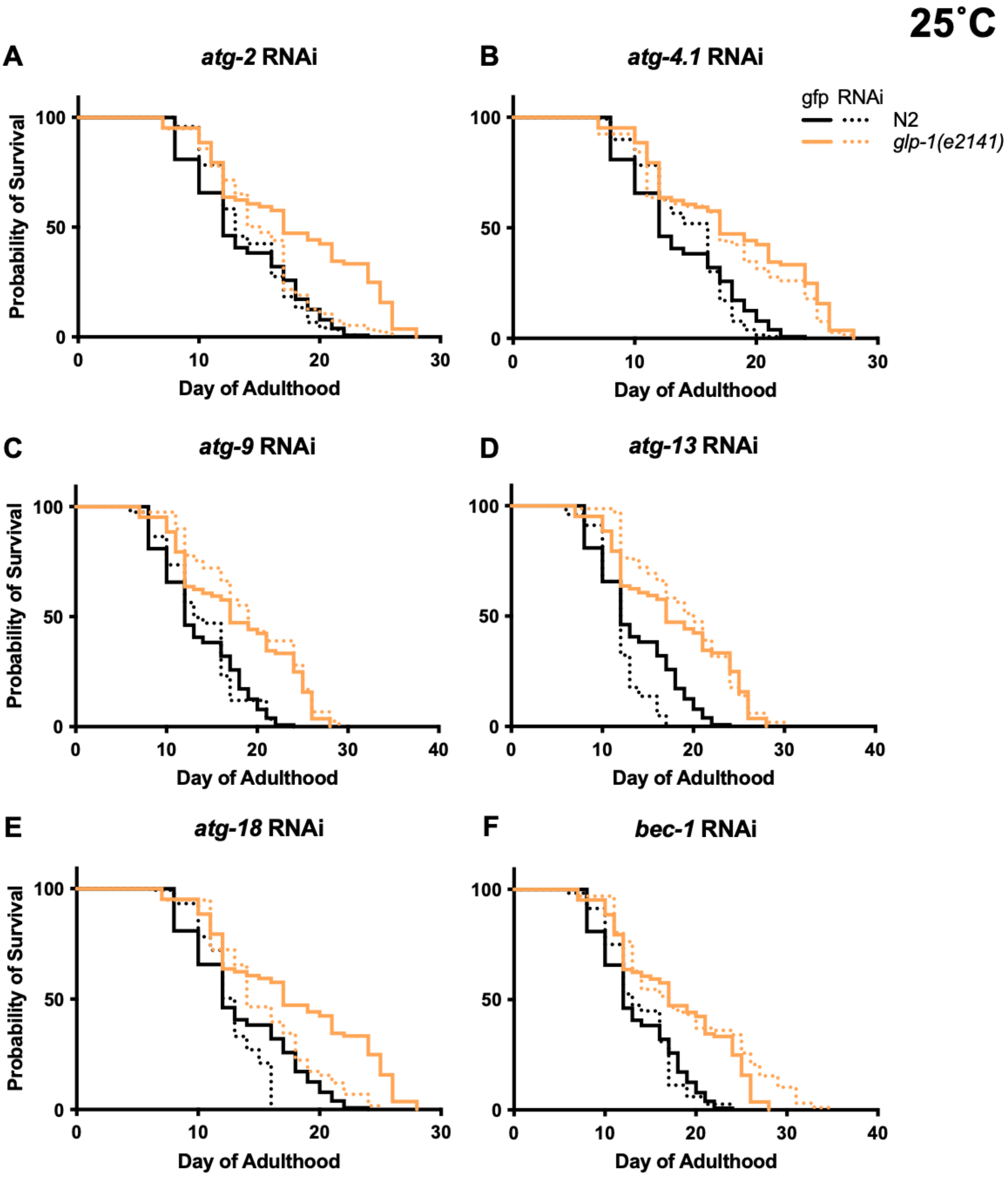

Supplementary Fig. 2. Effects of individual *atg* gene RNAi on N2 (wild type) and *glp-1(e2141)* lifespan (25˚C). Summed data, *N* = 2; for individual trials, see Table S7.

bioRxiv preprint doi: https://doi.org/10.1101/2025.07.28.667102; this version posted July 31, 2025. The copyright holder for this preprint (which was not certified by peer review) is the author/funder, who has granted bioRxiv a license to display the preprint in perpetuity. It is made available under aCC-BY 4.0 International license.

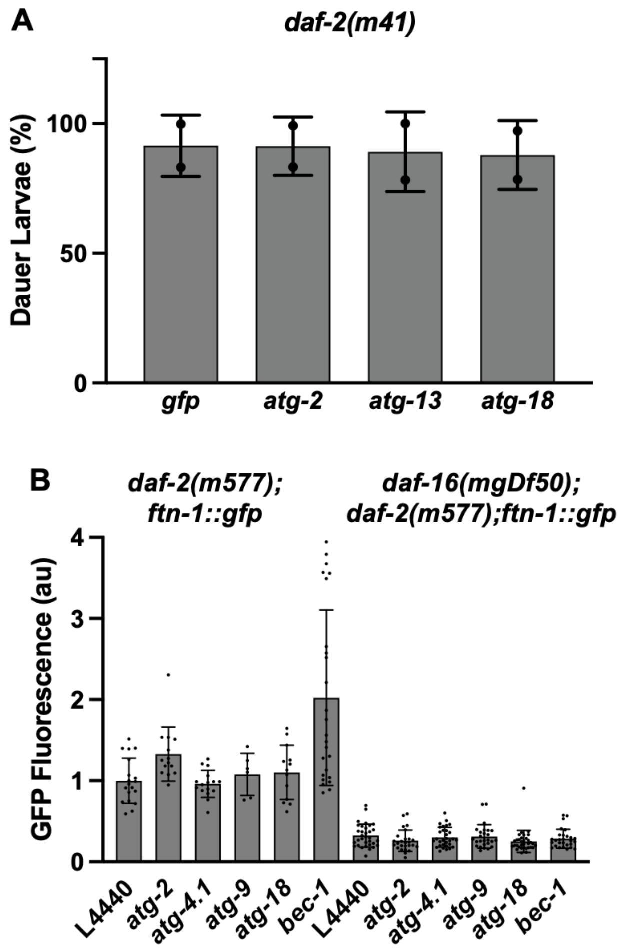

Supplementary Fig. S3. Tests for effects of *atg* RNAi on *daf-16*. (A) No suppression of *daf-2* Daf-c by *atg* RNAi. A sensitive Daf-c suppression assay was employed, using the class 1 mutant *daf-2(m41)* at 22.5˚C, previously found to form ~75% dauers [1]. Larval progeny of animals maintained for two generations on RNAi were examined. (B) No effects of *atg* RNAi on *daf-16* target gene expression. The reporter tested was based on the gene *ftn-1* (ferritin) [2].

Supplementary Table 1: Previous reports of effects of inhibition of autophagy on *daf-2* Age

C. elegans genotype	*atg* gene	Mammalian gene	Form of gene inhibition	Age at initiation of RNAi	Temperature	FUDR concentration (mM)	Change in *daf-2* mean LS cf RNAi control (p, log rank)[1]	Change in *daf-2(+)* control mean LS cf RNAi control (p, log rank)[1]	Greater reduction in LS in *daf-2*? (Cox proportional hazard)	Source
daf-2(e1370)	*bec-1*	*BECN1*	RNAi by microinjection	Injection of previous generation	15˚C	0	-46.6% (median lifespan) ($p < 0.001$)	-14.2% (median lifespan) (no statistics)	Yes ($p < 0.001$)	[3]
daf-2(e1370)	*atg-7*	*ATG7*	RNAi by feeding	Young adult	15˚C	0	Slight reduction (data not shown)	No effect (data not shown)	Not reported	[4]
daf-2(e1370)	*atg-7*	*ATG7*	RNAi by feeding	Young adult of previous generation (RNAi during development)	15˚C	0	-27.7% ($p = 5.4 \times 10^{-9}$)	-28.5% ($p = 4.7 \times 10^{-9}$)	Effect "appeared similar" (no statistical test)	[4]
daf-2(e1370)	*bec-1*	*BECN1*	RNAi by feeding	Young adult of previous generation (RNAi during development)	15˚C	0	Fig. 1A suggests no reduction in mean lifespan (no statistics)	Fig. 1B suggests a modest reduction in lifespan (no statistics)	Not reported	[4]
daf-2(e1370)	*lgg-3* (formerly *atg-12*)	*ATG12*	RNAi by feeding	Young adult	15˚C	0	Slight reduction (data not shown)	No effect (data not shown)	Not reported	[4]
daf-2(e1370)	*lgg-3* (formerly *atg-12*)	*ATG12*	RNAi by feeding	Young adult of previous generation (RNAi during development)	15˚C	0	-38.8% ($p = 2.1 \times 10^{-13}$)	-35.7% ($p = 1.1 \times 10^{-7}$)	Yes, $p = 0.129$ (whole populations), but $p = 0.0169$ (last 50% survivors)	[4]
fer-15(b26); daf-2(mu150); fem-1(hc17)	*bec-1*	*BECN1*	RNAi by feeding	Young adult	20˚C	0	Reduced lifespan in 6 trials ($p < 0.05$ in all cases)	No reduction in N2 lifespan in 3 trials (NS)	Yes	[5]
fer-15(b26); daf-2(mu150); fem-1(hc17)	*vps-34*	*PIK3C3*	RNAi by feeding	Young adult	20˚C	0	Reduced lifespan in 3 trials ($p < 0.05$ in all cases)	No reduction in N2 lifespan in 3 trials (NS)	Yes	[5]
daf-2(e1370)	*atg-9*	*ATG9A/ATG9B*	RNAi by feeding	Not specified	25˚C	1.21 M FUDR on day 1 of adulthood only	-17.5% (no statistical test)	Not reported	Unknown	[6]
daf-2(e1370)	*atg-18*	*WIPI1/WIPI2*	Mutation, *atg-18(gk378)*	N/A	25˚C	1.21 M FUDR on day 1 of adulthood only	-24.6% (no statistical test)	-23.2% ($p < 0.001$)	Unlikely (no statistical test)	[6]

daf-2 RNAi	*atg-18*	*WIPI1/WIPI2*	Mutation, *atg-18(gk378)*	L2 larval stage	25˚C	1.21 M FUDR on day 1 of adulthood only	-17.8% (no statistical test)	-23.2% ($p < 0.001$)	No	[6]
daf-2(e1370)	*bec-1*	*BECN1*	Mutation, *bec-1(ok691); Ex[bec-1(+)]*	N/A	25˚C	1.21 M FUDR on day 1 of adulthood only	-18.6% (no statistical test)	-26.4% ($p < 0.002$)	No	[6]
daf-2(e1370)	*lgg-1*	*GABARAP*	RNAi by feeding	Not specified	25˚C	1.21 M FUDR on day 1 of adulthood only	-8.2% (no statistical test)	Not reported	Unknown	[6]
daf-2(e1370)	*atg-3*	*ATG3*	RNAi by feeding	Maternal RNAi	20˚C	800 mM	**118.3** ($p = 0.012$)	81.4% ($p = 0.0026$)	No	[7]
daf-2(e1370)	*atg-4.1*	*ATG4A/ATG4B*	RNAi by feeding	Maternal RNAi	20˚C	800 mM	100.3 (NS)	79.9% ($p < 0.0001$)	No	[7]
daf-2(e1370)	*atg-4.2*	*ATG4C/ATG4D*	RNAi by feeding	Maternal RNAi	20˚C	800 mM	100.0 (NS)	92.9% ($p = 0.035$)	No	[7]
daf-2(e1370)	*atg-5*	*ATG5*	RNAi by feeding	Maternal RNAi	20˚C	800 mM	**126.4** ($p = 0.0014$)	80.8% ($p = 0.0001$)	No	[7]
daf-2(e1370)	*atg-7*	*ATG7*	RNAi by feeding	Maternal RNAi	20˚C	800 mM	**124.9** ($p = 0.0005$)	103.7% (NS)	No	[7]
daf-2(e1370)	*atg-9*	*ATG9A/ATG9B*	RNAi by feeding	Maternal RNAi	20˚C	800 mM	**121.2** ($p = 0.014$)	92.9% (NS)	No	[7]
daf-2(e1370)	*atg-10*	*ATG10*	RNAi by feeding	Maternal RNAi	20˚C	800 mM	107.5 (NS)	96.3% (NS)	No	[7]
daf-2(e1370)	*atg-16.2*	*ATG16L1/ ATG16L2*	RNAi by feeding	Maternal RNAi	20˚C	800 mM	**117.8** ($p = 0.0084$)	92.3% (NS)	No	[7]
daf-2(e1370)	*atg-18*	*WIPI1/WIPI2*	RNAi by feeding	Maternal RNAi	20˚C	800 mM	64.2 ($p = 0.0011$)	52.2% ($p < 0.0001$)	No	[7]
daf-2(e1370)	*bec-1*	*BECN1*	RNAi by feeding	Maternal RNAi	20˚C	800 mM	76.8 ($p = 0.021$)	84.0% ($p = 0.011$)	No	[7]
daf-2(e1370)	*lgg-1*	*GABARAP*	RNAi by feeding	Maternal RNAi	20˚C	800 mM	101.3 (NS)	87.7% ($p = 0.0012$)	No	[7]
daf-2(e1370)	*lgg-2*	*LC3*	RNAi by feeding	Maternal RNAi	20˚C	800 mM	110.3 (NS)	100.0% (NS)	No	[7]
daf-2(e1370)	*lgg-3*	*ATG12*	RNAi by feeding	Maternal RNAi	20˚C	800 mM	**125.6** ($p = 0.0027$)	97.2% (NS)	No	[7]
daf-2(e1370)	*unc-51*	*ULK1/UKL2*	RNAi by feeding	Maternal RNAi	20˚C	800 mM	**114.8**[2] ($p = 0.032$)	100.2% (NS)	No	[7]
daf-2(e1370)	*atg-3*	*ATG3*	RNAi by feeding	Early adulthood	20˚C	800 mM	101.1 (NS)	99.8 (NS)	No	[7]
daf-2(e1370)	*atg-4.1*	*ATG4A/ATG4B*	RNAi by feeding	Early adulthood	20˚C	800 mM	91.4 ($p = 0.022$)	96.1 (NS)	Possible (no statistical test)	[7]
daf-2(e1370)	*atg-4.2*	*ATG4C/ATG4D*	RNAi by feeding	Early adulthood	20˚C	800 mM	97.8 (NS)	94.2 (NS)	No	[7]
daf-2(e1370)	*atg-5*	*ATG5*	RNAi by feeding	Early adulthood	20˚C	800 mM	99.3 (NS)	102.2 (NS)	No	[7]
daf-2(e1370)	*atg-7*	*ATG7*	RNAi by feeding	Early adulthood	20˚C	800 mM	97.5 (NS)	**107.6** ($p = 0.020$)	No	[7]
daf-2(e1370)	*atg-9*	*ATG9A/ATG9B*	RNAi by feeding	Early adulthood	20˚C	800 mM	**115.8** ($p = 0.0013$)	**111.4** ($p = 0.0013$)	No	[7]
daf-2(e1370)	*atg-10*	*ATG10*	RNAi by feeding	Early adulthood	20˚C	800 mM	97.0 (NS)	98.0 (NS)	No	[7]

daf-2(e1370)	*atg-16.2*	*ATG16L1/ ATG16L2*	RNAi by feeding	Early adulthood	20˚C	800 mM	101.4 (NS)	103.4 (NS)	No	[7]
daf-2(e1370)	*atg-18*	*WIPI1/WIPI2*	RNAi by feeding	Early adulthood	20˚C	800 mM	95.7 (NS)	77.1 (p = 0.0010)	No	[7]
daf-2(e1370)	*lgg-1*	*GABARAP*	RNAi by feeding	Early adulthood	20˚C	800 mM	96.7 (NS)	89.5 (p = 0.019)	No	[7]
daf-2(e1370)	*lgg-2*	*LC3*	RNAi by feeding	Early adulthood	20˚C	800 mM	106.2 (NS)	105.3 (NS)	No	[7]
daf-2(e1370)	*lgg-3*	*ATG12*	RNAi by feeding	Early adulthood	20˚C	800 mM	98.6 (NS)	108.1 (NS)	No	[7]
daf-2(e1370)	*bec-1*	*BECN1*	RNAi by feeding	Early adulthood	20˚C	800 mM	**111.4** (p = 0.0029)	**110.1** (p = 0.028)	No	[7]
daf-2(e1370)	*unc-51*	*ULK1/UKL2*	RNAi by feeding	Early adulthood	20˚C	800 mM	**111.0** (p = 0.010)	**109.4** (p = 0.024)	No	[7]
daf-2(e1370)	*atg-18*	*WIPI1/WIPI2*	RNAi by feeding	Early adulthood	20˚C	0	-56, -51, -51, -52, -52 (p < 0.0001)	Test not performed	Insufficient data	[8]
daf-2(e1370	*lgg-1*	*GABARAP*	RNAi by feeding	Early adulthood	20˚C	0	-21, -22 (p < 0.0001)	Test not performed	Insufficient data	[8]
daf-2(e1370)	*atg-18*	*WIPI1/WIPI2*	Mutation, *atg-18(gk378)*	N/A	20˚C	0	61, 75, 71 (median lifespan) (p < 0.0001)	50, 67, 68 (median lifespan) (p < 0.0001)	Possible trend (no statistical test)	[9]
rrf-3(pk1426); daf-2(e1370)	*bec-1*	*BECN1*	RNAi by feeding	Day 10 of adulthood[2]	20˚C	0	**21.3, 11.3, 17.7** (p < 0.0001)	**39.3, 51.9, 62.5** (p < 0.0001)	No	[10]
daf-2 RNAi (neuron limited)	*atg-16.2*	*ATG16L1/ ATG16L2*	Mutation, *atg-16.2; sid-1; rgef-1p::sid-1 + unc-122p::rfp*	Not stated	20˚C	0	**95.4**, **95.4**, **50.3** (p < 0.0001)	Test not performed	No	[11]

[1]Bold, significant increase in lifespan after autophagy pathway gene RNAi.

[2]Day 0 defined as L4 stage.

Supplementary Table 6: Previous reports of effects of inhibition of autophagy on GSC(-) Age

***C. elegans* genotype**	***atg* gene**	**Mammalian gene**	**Form of gene inhibition**	**Age at initiation of RNAi**	**Temperature**	**FUDR concentration (mM)**	**Change in GSC(-) mean lifespan relative to RNAi control (p, log rank)**	**Change in N2 control mean lifespan relative to RNAi control (p, log rank)**	**Greater reduction in lifespan in GSC(-)?**	**Source**
glp-1(e2141)	*bec-1*	*BECN1*	RNAi by feeding	D1 of adulthood	25˚C until D1, then 20˚C	0	-22%, -17%, -21% (p < 0.0001, 0.0005, < 0.0001)	7%, 0,% 7% (p = 0.14, 0.94, 0.27)	Yes	[12]
glp-1(e2141)	*lgg-1*	*GABARAP*	RNAi by feeding	D1 of adulthood	25˚C until D1, then 20˚C	0	-18%, -18%, -39% (p = 0.009, < 0.0001, < 0.0001)	1%, 9%, -2% (p = 0.89, 0.06, 0.30)	Yes	[12]
glp-1(e2141)	*unc-51*	*ULK1/UKL2*	RNAi by feeding	D1 of adulthood	25˚C until D1, then 20˚C	0	-27%, -11%, -17% (p < 0.0001, 0.040, 0.0028)	2%, 10%, -2% (p = 0.90, 0.068, 0.67)	Yes	[12]
glp-1(e2141)	*vps-34*	*PIK3C3*	RNAi by feeding	D1 of adulthood	25˚C until D1, then 20˚C	0	-25%, -26%, -24% (p < 0.0001)	6%, -2%, -5% (p = 0.19, 0.27, 0.52)	Yes	[12]
glp-1(e2141)	*atg-18*	*WIPI1/WIPI2*	RNAi by feeding	D1 of adulthood	25˚C until D1, then 20˚C	0	-35%, -33%, -28% (p < 0.0001)	6%, -3%, 10% (p = 0.38, 0.52, 0.85)	Yes	[12]
mes-1(bn17)	*vps-34*	*PIK3C3*	RNAi by feeding	D1 of adulthood	25˚C until D1, then 20˚C	0	-31% (p < 0.0001)	2% (p = 0.96)	Yes	[12]
mes-1(bn17)	*atg-18*	*WIPI1/WIPI2*	RNAi by feeding	D1 of adulthood	25˚C until D1, then 20˚C	0	-30% (p < 0.0001)	3% (p = 0.60)	Yes	[12]
glp-1(e2141)	*lmp-1*	*LAMP-1*	RNAi by feeding	D1 of adulthood	25˚C until D1, then 20˚C	0	-11%, -4%, -11% (p = 0.069, 0.58, 0.041)	1%, -12%, 13% (p = 0.79, 0.0059, 0.021)	Possibly	[13]
glp-1(e2141)	*vha-16*	*ATP6V0D1*	RNAi by feeding	D1 of adulthood	25˚C until D1, then 20˚C	0	-47%, -42%, -32% (p <0.0001)	-11%, -16%, -9% (p = 0.0015, 0.0001, 0.011)	Yes	[13]
glp-1(e2141)	*bec-1*	*BECN1*	RNAi by feeding	D10 of adulthood[2]	25˚C until D1[2], then 20˚C	0	**24.0**, **34.9**, **32.5**, **39.0** (p < 0.0001)	Not performed	No	[10]

[1]Bold, significant increase in lifespan after autophagy pathway gene RNAi.
[2]Day 0 defined as L4 stage.

bioRxiv preprint doi: https://doi.org/10.1101/2025.07.28.667102; this version posted July 31, 2025. The copyright holder for this preprint (which was not certified by peer review) is the author/funder, who has granted bioRxiv a license to display the preprint in perpetuity. It is made available under aCC-BY 4.0 International license.

Supplementary references

1. Gems D, Sutton AJ, Sundermeyer ML, Larsen PL, Albert PS, King KV, et al. Two pleiotropic classes of *daf-2* mutation affect larval arrest, adult behavior, reproduction and longevity in *Caenorhabditis elegans*. Genetics. 1998; 150:129-55.
2. Ackerman D, Gems D. Insulin/IGF-1 and hypoxia signaling act in concert to regulate iron homeostasis in *C. elegans*. PLoS Genet. 2012; 8:e1002498.
3. Meléndez A, Tallóczy Z, Seaman M, Eskelinen E-L, Hall DH, Levine B. Autophagy genes are essential for dauer development and life-span extension in *C. elegans*. Science. 2003; 301:1387-91.
4. Hars ES, Qi H, Ryazanov AG, Jin S, Cai L, Hu C, et al. Autophagy regulates ageing in *C. elegans*. Autophagy. 2007; 3:93-5.
5. Hansen M, Chandra A, Mitic LL, Onken B, Driscoll M, Kenyon C. A role for autophagy in the extension of lifespan by dietary restriction in *C. elegans*. PLoS Genet. 2008; 4:e24.
6. Toth ML, Sigmond T, Borsos E, Barna J, Erdelyi P, Takacs-Vellai K, et al. Longevity pathways converge on autophagy genes to regulate life span in *Caenorhabditis elegans.* Autophagy. 2008; 4:330-8.
7. Hashimoto Y, Ookuma S, Nishida E. Lifespan extension by suppression of autophagy genes in *Caenorhabditis elegans*. Genes Cells. 2009; 14:717-26.
8. Chang J, Kumsta C, Hellman A, Adams L, Hansen M. Spatiotemporal regulation of autophagy during *Caenorhabditis elegans* aging. eLife. 2017; 6:e18459.
9. Minnerly J, Zhang J, Parker T, Kaul T, Jia K. The cell non-autonomous function of ATG-18 is essential for neuroendocrine regulation of *Caenorhabditis elegans* lifespan. PLoS Genet. 2017; 13:e1006764.
10. Wilhelm T, Byrne J, Medina R, Geisinger J, Hajduskova M, Tursun B, et al. Neuronal inhibition of the autophagy nucleation complex extends life span in post-reproductive *C. elegans* Genes Develop. 2017; 31:1561–72.
11. Yang Y, Arnold ML, Lange CM, Sun LH, Broussalian M, Doroodian S, et al. Autophagy protein ATG-16.2 and its WD40 domain mediate the beneficial effects of inhibiting early-acting autophagy genes in *C. elegans* neurons. Nat Aging. 2024; 4:198-212.
12. Lapierre L, Gelino S, Meléndez A, Hansen M. Autophagy and lipid metabolism coordinately modulate life span in germline-less *C. elegans*. Curr Biol. 2011; 21:1507-14.
13. Lapierre L, De Magalhaes Filho C, McQuary P, Chu C, Visvikis O, Chang J, et al. The TFEB orthologue HLH-30 regulates autophagy and modulates longevity in *Caenorhabditis elegans*. Nat Commun. 2013; 4:2267.
